# Expert analogy use in a naturalistic setting

**DOI:** 10.3389/fpsyg.2014.01333

**Published:** 2014-11-26

**Authors:** Donald R. Kretz, Daniel C. Krawczyk

**Affiliations:** ^1^School of Behavioral and Brain Sciences, The University of Texas at DallasRichardson, TX, USA; ^2^Department of Psychiatry, University of Texas Southwestern Medical CenterDallas, TX, USA

**Keywords:** analogy, expertise, naturalistic, reasoning, problem-solving

## Abstract

The use of analogy is an important component of human cognition. The type of analogy we produce and communicate depends heavily on a number of factors, such as the setting, the level of domain expertise present, and the speaker's goal or intent. In this observational study, we recorded economics experts during scientific discussion and examined the categorical distance and structural depth of the analogies they produced. We also sought to characterize the purpose of the analogies that were generated. Our results supported previous conclusions about the infrequency of superficial similarity in subject-generated analogs, but also showed that distance and depth characteristics were more evenly balanced than in previous observational studies. This finding was likely due to the nature of the goals of the participants, as well as the broader nature of their expertise. An analysis of analogical purpose indicated that the generation of concrete source examples of more general target concepts was most prevalent. We also noted frequent instances of analogies intended to form visual images of source concepts. Other common purposes for analogies were the addition of colorful speech, inclusion (i.e., subsumption) of a target into a source concept, or differentiation between source and target concepts. We found no association between depth and either of the other **two** characteristics, but our findings suggest a relationship between purpose and distance; i.e., that visual imagery typically entailed an outside-domain source whereas exemplification was most frequently accomplished using within-domain analogies. Overall, we observed a rich and diverse set of spontaneously produced analogical comparisons. The high degree of expertise within the observed group along with the richly comparative nature of the economics discipline likely contributed to this analogical abundance.

## Introduction

The importance of analogy has been described in many ways by notable researchers. Polya (1957) wrote that analogy “pervades all our thinking,” Holyoak et al. (2001) called it “a central component of human cognition,” and Hofstadter (2001) referred to it as “the lifeblood … of human thinking.” Because of its perceived importance in cognitive functioning, the use of analogy in thought and language has been studied extensively in cognitive psychology, cognitive development, and cognitive science since the early 1980s. Research examining analogical production and retrieval under experimental conditions has provided a wealth of valuable data. Far fewer studies of these important phenomena have been conducted in naturalistic settings.

The importance of studying analogical production “in the wild” was emphasized by one of its most prominent researchers. Just as it is necessary to conduct both *in vivo* and *in vitro* studies to fully understand biological phenomena, Dunbar (1995, 2001) argued that it is likewise necessary to conduct both naturalistic and experimental studies in cognitive research to fully understand the cognitive processes involved in reasoning and analysis. He modeled his approach after techniques applied in biological research, referring to this paradigm as “*in vivo/in vitro*.” Observing behavior in naturalistic settings provides several advantages: (a) behaviors are unconstrained by laboratory conditions and are not instigated by artificial or experimental stimuli, (b) the setting emphasizes processes rather than outcomes, and (c) there is a clear relationship between observed behaviors and the domain of interest (Dunbar, 1995; Crano and Brewer, 2002). These conditions are particularly important when investigating analogical thinking which involves linking one's current context with prior knowledge in a spontaneous fashion. It is important to note, however, that the observational approach lacks the superior structure and clarity of laboratory-based experiments.

Because there are many commonly used definitions of the term *analogy*, we felt it necessary to offer a clear definition that captures the essential characteristics applied by researchers in this field. A frequently used means of conveying an understanding of an unfamiliar concept is by drawing a comparison to similar, more familiar concepts. An analogy conveys more than literal similarity between two objects or concepts (Gentner, 1983). As a process, analogy involves the search for and selection of a well-understood *source* from long-term memory, followed by the transfer of meaning from that source to a less familiar *target* (Spellman and Holyoak, 1996). The set of correspondences between a source and target are referred to as a *source-target mapping*. In contrast to other forms of likeness or similarity, analogy is based on a comparison of structural relations, or systems of relations, rather than a mere resemblance of surface properties or attributes (Gentner and Markman, 1997). A system of relations can be quite complex, and all of its mappings may not be apparent. Some evidence suggests, however, that analogical mapping is not entirely relational (Ball et al., 2004; Bearman et al., 2007).

In the current study, we applied Dunbar's *in vivo* approach to observe and analyze some of the characteristics of source analogs produced during live, open discussion involving problems in economics and discuss some of the factors influencing their production. We chose a behavioral economics group to observe, as economists seek to broadly explain complex real-world human behavior in the context of models, games, and examples. All of these techniques rely heavily upon comparisons between different states of the world, different types of behavior, and combinations of models and situations. Many of these comparisons are potentially analogical, drawing heavily upon the expertise of the individual and the broader group. Data were collected from the weekly sessions of an economics reading group, which consisted of participants whose expertise in the broad field of economics varied in terms of depth and academic specialization.

### Analogical depth

One of the factors that has been shown to influence source retrieval is the level, or *depth*, of similarity between a target and the chosen source. Past research has shown that source-to-target mapping is primarily driven by the comparison of structural relations between source and target concepts but retrieval may be facilitated by superficial characteristics (Gick and Holyoak, 1980, 1983; Gentner et al., 1993; Forbus et al., 1997). In other words, people will rely on structural details to find appropriate sources when mapping an analogy, but perhaps find it easier to rely on overlapping surface features in addition to underlying structure when retrieving a correspondence (Holyoak and Koh, 1987; Holyoak and Thagard, 1995; Dunbar, 2001). The use of superficial characteristics for comparison is particularly apparent when source and target analogs are generated a priori and provided to an individual, who must then consider the nature of the relationship or relationships.

When people generate their own analogies by drawing upon their own knowledge, research has shown that fewer superficial similarities between analogs are observed. Studies by Dunbar (1995, 1997) and Blanchette and Dunbar (1997, 2000, 2001) revealed that more than half of the analogies produced during biology laboratory meetings and political discourse, respectively, showed no apparent surface similarity. The Bearman et al. (2007) management study showed that 73% of analogies were only structural in nature. With this in mind, the first goal of this study was to examine the depth of analogies produced by economics experts during scientific discourse. Results were expected to show infrequent surface feature overlap and provide additional support for earlier findings.

### Analogical distance

A second important feature in analogy use concerns the range, or *distance*, between the domains of the source and target analogs. Dunbar (1995) defined three categories of analogy in terms of their degree of domain separation: (a) *long-distance* describes a source drawn from a very different domain (also referred to as *outside-domain*, or *out-of-category*), (b) *regional* refers to a source mapped from a similar domain (e.g., economics to finance or public administration), and (c) *local* maps a target to a source in the same domain. Both local and regional classes are collectively referred to as *within-domain*, or *within-category*. Because of the subjective nature of judging domain distance between similar domains, many observational studies simply categorize analogies using a binary choice of within-category or outside-category. Analogies formed within the same category might compare biological organisms or investment strategies, while commonly referenced domains for outside-category analogies include sports (e.g., one might fail and “strike out at the plate” or succeed and “push the ball over the goal line”) and the supernatural (e.g., “it works so well that it's like magic”).

Research on the domain separation of analogies has provided contrasting results. Dunbar's studies of scientific reasoning set in microbiology laboratories showed heavy use of within-category analogies—98% of the analogies generated were classified as either local or regional (Dunbar, 1995, 1997). A study by Saner and Schunn (1999) produced a similar but narrower finding—researchers in psychology laboratory meetings and colloquia made frequent use of analogies, and more than 75% of them were within the same domain. In contrast, Blanchette and Dunbar found that 77% of the analogies that appeared in political articles aimed at more general audiences were outside-category (2001). Christensen and Schunn's, 2007 engineering design group study showed a more balanced mix of analogies—55% were within-category while 45% were outside-category.

One explanation for the difference in domain distance is that the collective expertise of the audience may influence the selection of analogy. When addressing other domain experts with specialized knowledge, within-category analogs may prove more effective whereas an outside-domain analogy might be more attractive choice for a general audience. The type of task or function performed by the analogizer is another possible constraint. Our second goal was to observe the use of within- and outside-domain analogies produced by the economics experts. We expected to find a fairly balanced use of both styles, which differs somewhat from earlier findings. Our expectation was motivated by the different subdomains and varying levels of subject expertise within the reading group. Furthermore, in light of the stated belief that within-domain analogies tend to involve a higher degree of superficial similarity than outside-domain analogies, we investigated whether such a relationship emerged.

### Analogical purpose

Thirdly, past research has shown that the goal of the individual producing the analogy is likely to influence the process of selection and transfer (Spellman and Holyoak, 1996). Prior observational studies have examined the types of goals that emerge from the production of analogies, and the goals themselves tend to be highly domain-specific and task-specific. For example, the four goals derived from the microbiology laboratories by Dunbar (1995, 1997) were: forming hypotheses, designing experiments, modifying experiments, and explaining issues and concepts to other scientists. The management decision-making study by Bearman et al. (2002) identified only two goals: problem-solving and illustration. Similarly, the Christensen and Schunn (2007) study of analogizing during engineering design meetings reported three functions: identifying problems, solving problems, and explaining concepts. Our economics reading group differed in its function from the settings of the studies mentioned above. The purpose of the group was limited to explaining and understanding experiments performed by other researchers; i.e., the experts in our observational study critiqued experimental designs and analyzed results.

Blanchette and Dunbar (2001) provided evidence that goals influenced the choice of analogy. When supporting a favorable position, emotionally positive analogies were more commonly chosen over those displaying negative emotional ideas. Conversely, when criticizing an unfavorable position, emotionally negative analogies were more frequent. Because analogical retrieval appears subject to the influence of the purpose for which it was produced, our third goal was to analyze the range of goals that emerge from the use of analogies by the economics group and their potential effect on analogical distance and depth.

## Methods

### Participants

The setting for this study was the School of Economic, Political, and Policy Sciences (EPPS) weekly reading group at The University of Texas at Dallas during the Spring semester of 2011. Participants included male and female reading group attendees. The average weekly attendance was approximately 20 participants, which included both faculty members (~25%) and graduate students (~75%). The group was largely the same from week to week, but attendance did fluctuate and not all participants attended regularly. Participants' academic expertise was mixed, and included sub-disciplines such as econometrics, experimental economics, and game theory. A few of the attendees had no exposure to experimental methods.

The sessions were conducted in typical reading group fashion. One of the senior faculty members acted as the group's moderator, and one student participant was assigned each week to lead the following week's discussion of one or more chosen research papers. Following a brief presentation of the paper by the assigned student, a free and open discussion followed in which the members of the group examined and dissected the experimental methods and results described in the paper.

### Procedure

#### Session recordings

The investigator attended and made audio recordings at each of five group meetings, but did not participate in the discussions and appeared to have minimal impact on group interaction. Group members understood that their discussions would be evaluated (the moderator referred to it as “the science behind the science”). The research goal of examining the spontaneous use of analogies was not revealed, however. Although each session was scheduled to last for 90 min, actual discussion times ranged from 65 to 110 min. In all, approximately 7 h of discourse were recorded.

#### Transcription procedure

Four transcribers participated in the initial processing of the discourse—the primary author and three undergraduate Psychology students. Only the primary author and one of the undergraduates had any significant transcription experience prior to the task. None of the undergraduate transcribers had exposure to Economics beyond introductory coursework. Transcribers were given instruction by the primary author on what constitutes an analogy based on the definitions presented earlier in this paper, then solved practice problems on recognizing analogies from non-analogies by identifying sources and targets. The period of instruction lasted approximately 30 min.

For purposes of indoctrination, each undergraduate transcriber was given one of the sessions for practice and directed to process at least 30 min of the recording. They were instructed to transcribe passages in which a possible analogy was made, taking care to include all of the important source and target information. Their instruction was: “when in doubt, include it.” The primary author evaluated their performance and made individual adjustments until the results were satisfactory and consistent.

To address the possibility of subtle, easily-overlooked analogies, every audio recording was processed by two transcribers. To be included for analysis, a passage needed only to appear on either transcriber's log, not both. In order to be missed, a passage would have to have been heard by both transcribers and rejected.

#### Coding procedure

The two authors performed the coding duties. Both were experienced coders with strong knowledge of analogy literature and considerable research experience. Each of the transcribed segments was first evaluated for the presence of one or more analogies based on the definition stated earlier. Comparisons based only on literal similarities were considered non-analogies. If a segment was judged to contain no analogy, no further evaluation was performed. If an analogy was deemed present, the source, target, source domain, and target domain of the analogy were recorded. Each analogy was then coded along the three dimensions of distance, depth, and purpose.

Both coders rated the entire set and reliability was calculated for each dimension. Distance was rated using the commonly applied *within-domain* and *outside-domain* categories. Following the example of Saner and Schunn (1999) in which the authors collapsed all psychology-related categories into “within-domain,” we likewise considered all analogies related to economics, finance, statistics, probabilities, and game theory to be in the same domain. Depth was rated as superficial if the source and target shared surface characteristics; if not, it was rated as structural. Two passes were made by each coder to rate the purpose. The first pass was used to generate and agree on a list of functions represented by the set of analogies. Then, the coders used the list from the first pass as set of categories for rating analogies in the second pass.

## Results

### Segment transcription and analogy extraction

Transcription of the five recorded sessions yielded 114 unique passages with possible analogies (*M* = 16.29 analogy segments per hour). See Appendix A for a sampling of extracted passages. When judging whether a passage contained an analogy, the coders agreed on 96% (*n* = 109) of the 114 passages. Of these, most were rated as containing an analogy (*n* = 91, 83%). Passages lacking the basic ingredients of an analogy (i.e., source-target mapping and knowledge transfer) or showing evidence of a literal comparison (*n* = 18, 17%) were eliminated. Five passages were inconclusive and were likewise eliminated. The inter-rater reliability for identifying analogies was strong (κ = 0.85). Some of the passages were found to contain multiple analogies. In all, 97 analogies were extracted for the remainder of this analysis.

### Analogical depth

In coding for analogical depth, the coders agreed on 91% (*n* = 88) of the 97 analogies. Of these, analogies showing no obvious overlap in surface characteristics (*n* = 69, 78%) far outnumbered those where superficial similarities were present (*n* = 19, 22%). The inter-rater reliability was found to be substantial (κ = 0.75).

### Analogical distance

In coding for analogical distance, the coders agreed on 86% (*n* = 83) of the 97 analogies. Of these, within-domain (*n* = 44, 53%) and outside-domain (*n* = 39, 47%) analogies were almost evenly distributed. The inter-rater reliability was again found to be substantial (κ = 0.71).

### Analogical purpose

At stated above, we did not impose a priori categorical restrictions on coding for analogical purpose. Rather, the coders were free to make subjective judgments as to the intent of the speaker on the first pass through the data. From these impressions, we grouped similar items and derived a set of categories for coding the analogies on the second pass. The derived taxonomy of rating categories was as follows: Differentiation (highlighting differences between source and target), Inclusion (indicating that the target was a type or component of the source), Example (indicating that the target was an instance of the source concept), Visualization (intended to create a picture or image in the mind of the audience), Emotion (appealing to feelings of the audience, or drawing on the emotion of the expert), Critique (using the source to point out shortcomings in the target), Exaggeration (gratuitous use of colorful phrasing), and Abstraction (broaden the target concept using a more general source concept). Where an analogy plausibly served multiple purposes, the raters chose the strongest.

In coding for analogical purpose, the coders agreed on 79% (*n* = 77) of the 97 analogies. Examples were the most prevalent (*n* = 27, 35%), followed by visualizations (*n* = 19, 25%). A fair number of exaggerations (*n* = 11, 14%), inclusions (*n* = 9, 12%), and differentiations (*n* = 8, 10%) were observed, but abstractions (*n* = 2, 3%) and critiques (*n* = 1, 1%) were uncommon. The inter-rater reliability for analogical purpose was substantial (κ = 0.74).

### Association between depth and distance

As reported above, most of the analogies were rated as structural analogies. Of those, there was an even distribution of outside-domain (*n* = 31, 50%) and within-domain (*n* = 31, 50%) analogies. The superficial analogies were likewise split between outside-domain (*n* = 7, 54%) and within-domain (*n* = 6, 46%). It was determined that there was no significant distance-depth association [χ^2^_(1,*N* = 75)_ = 0.06, *p* = 0.80], suggesting that the two variables are independent.

### Association between depth and purpose

Almost half of the analogies were rated as either structural-example (*n* = 18, 25%) or structural-visualization analogies (*n* = 16, 23%). Though the distribution of purpose ratings was highly skewed, it was skewed similarly over both categories of analogical depth and no significant depth-purpose association was found [χ^2^_(7,*N* = 71)_ = 8.74, *p* = 0.27]. This finding suggests that these two variables are also independent.

### Association between distance and purpose

In contrast to the two findings above, an examination of analogical distance as a function of the speaker's purpose did reveal a significant association [χ^2^_(7,*N* = 66)_ = 34.94, *p* < 0.005]. When a speaker sought to produce an analogy that created a visual image, an out-of-domain source was typically selected (*n* = 16, 24%). On the other hand, when an analogizing by example, a source-target transfer from within the same domain was most commonly observed (*n* = 18, 27%).

## Discussion

The most prominent observation to come from this study was that the use of analogy for exploration and explanation among economics experts and students was rich and abundant. In 427 min of discourse, 97 analogies were extracted, suggesting that analogies are both commonly used and serve as an important component in human reasoning and in understanding problems. The reading group setting enabled the observation of actual experts spontaneously forming analogies using their semantic knowledge applied to economics, a domain likely to entail more freedom to move between and among domains of knowledge than the previously investigated biology and political domains. The selection and fluid assembly of analogies during discourse may help to reveal the core principles involved in analogical thinking among experts. This study's findings will be discussed in the context of prior evidence.

### Experts in the field of economics

We chose to study behavioral economics experts in this study. Economists seek to broadly explain complex real-world human behavior in the context of equations, models, games, and hypothetical examples. All of these techniques rely heavily upon comparisons between different states of the world, different types of behavior, and combinations of models and situations. While some feature or object-based similarity occurs in comparisons between economic models and real-world behavior, it can be argued that the majority of the comparisons are about relations and systems of relations. For example, in the economic Ultimatum Game (Guth et al., 1982), two individuals each make monetary decisions that will be reviewed and reacted to by the other. The Ultimatum Game can be compared to many situations in life in which banks, individuals, or nations either choose to cooperate or not with regard to money, goods, or military action. Thus, many of the comparisons used in behavioral economics are likely to be analogical and may occur with greater frequency than observed in laboratory settings. The expertise of the individuals involved in behavioral economics discussions and expertise levels of the broader group are likely to encourage potentially rich and relationally deep analogies.

Expertise was a critical element to this study, as the role of domain knowledge in analogy production is not yet well understood. The level of expertise among reading group participants varied, but all had sufficient knowledge to be considered experts in the field. In some observational studies, expertise was narrowly concentrated [Dunbar's (1995, 1997) molecular biology group], whereas in others, general audiences possessed little to no domain knowledge [e.g., Blanchette and Dunbar (2001) political news study]. In our economics reading group, the expertise was both deep and broad, covering a range of specializations such as experimental economics, econometrics, and game theory.

There is growing evidence that the depth of expertise of the audience may influence analogical selection. It is reasonable to hypothesize that the breadth of expertise may contribute, as well. Blanchette and Dunbar (2001) showed more frequent use of outside-domain analogies when the intended audience consisted of non-experts and correspondingly more prevalent within-domain analogies among fellow experts. Additionally, experts appear more likely to exploit the complex relational nature of analogies while avoiding the tendency to rely on superficial features for comparison. The findings of Bearman et al. (2007) suggested that experts tend to compare structural relationships in all activities but novices show differences; i.e., when engaged in problem solving activities, novices rely on structural analogies but incorporate superficial features in their comparisons when illustrating or explaining.

We based our depth and distance expectations for this study largely on these findings. We did, as expected, observe a more even distribution of distance in analogies and infrequent use of superficial features in making comparisons. Relative to novices, experts can draw on a great deal of accumulated knowledge when thinking and reasoning (Bearman et al., 2007). It stands to reason that they are better able to exploit the deep, structural nature of source information as a result. Non-experts, on the other hand, lack the same deep encoding of domain information and may need to rely more heavily on superficial characteristics. The evidence is inconsistent, however; the Blanchette and Dunbar (2000) study points to novice use of structure as well.

### Analogical depth—superficial vs. structural

Structural correspondences in the underlying system of relations between source and target elements represent an important component of analogies. Past experimental research demonstrated that superficial features influence the selection of source analogs (Gick and Holyoak, 1980, 1983; Gentner et al., 1993; Forbus et al., 1997), and studies in naturalistic settings suggest that other factors may contribute, as well (Blanchette and Dunbar, 2001; Bearman et al., 2007). The ratings from this study showed that the economics experts relied primarily on structural components of analogies (78%) with infrequent use of superficial feature comparisons (22%).

The data collected from the reading group sessions contained a dense set of complex comparisons rich in structure. Because of the unconstrained, yet guided, nature of the discussion, participants experienced a great deal of freedom to explore, compare, and explain complex target concepts. The reading group setting had certain features of prior story-based analogical reminding studies (Gentner et al., 1993; Wharton et al., 1994; Catrambone, 1997), as participants compared the contents of journal papers and the various experimental methods they described. The setting also had characteristics of the Blanchette and Dunbar “production paradigm” studies (2001) in which participants generated source analogs from their own knowledge and experiences. Perhaps not all settings are completely retrieval-based or production-based; rather, the degree to which the activity combines retrieval tasks with production tasks may determine the balance of analogical depth applied. Additionally, the occasional use of superficial comparisons likely reflects a tendency to spark heightened interest by making a link to a distant analogous domain during their descriptions of economic processes. Indeed, some of the surface-level analogies tabulated could be considered to be turns of phrase or metaphorical comparisons. The use of such comparisons can be helpful in making speech more interesting to the listener and to add points of common reference periodically.

### Analogical distance—within domain vs. outside domain

One possible explanation for the observed balance between within-category and out-of-category analogies was that subject matter expertise shared among a speaker and audience influenced source selection. Observational data offer support to this hypothesis. In Dunbar's (1995, 1997) biology laboratory study, scientists overwhelmingly produced within-domain analogies (98%). Saner and Schunn (1999) also observed high rates of within-domain analogies in their study of psychology laboratory meetings (81% within-domain) and colloquia (77% within-domain). In contrast, Blanchette and Dunbar (2001) studied opinion articles in the mainstream press, where the audience was the population at large. Here, they found that most analogies (77%) were from outside the target domain. Based on these findings, it appears that experts produce more within-domain analogies when communicating knowledge to their fellow subject matter experts, but generate analogies from sources from other domains when the audience consists of non-experts.

A second, but related, explanation stated that the goals of the participants were an influencing factor on source distance (Dunbar, 1995; Holyoak and Thagard, 1997; Blanchette and Dunbar, 2001). In Dunbar's biology lab study, the scientists were heavily focused on examining unexpected experimental results and resolving methodological problems. However, in the Dunbar and Blanchette studies involving both experts and non-experts, the experts' goals were to educate and/or persuade. In our study, there was no “discovery task”—i.e., there were no new hypotheses being generated, no new designs being developed, and no problems being solved. Rather, the discussion involved a great deal of comparison, both integrating and differentiating details of experimental methods and results. It may be that tasks involving a greater amount of creativity or innovation lead to more frequent use of same-domain analogies.

The results from the current study were more balanced. Out-of-category analogies were observed most frequently (53%), but within-category analogies accounted for a sizeable portion of the total as well (47%). These results fall squarely between the previous findings, but a plausible explanation can be made. If the number of within- and outside-domain analogies is a function of expertise, then the balance between them might be expected to vary with the range and depth of expertise. In the EPPS reading group, the participants all had some degree of expertise in economics, but their domain experience varied in terms of sub-discipline (e.g., econometrics, game theory, behavioral economics) and academic career longevity (i.e., faculty or graduate student). Hence, common expertise is likely to be an influencing factor, as Dunbar suggested, but the amount of influence it has on source distance may moderated by the variability in such factors as range and depth. Additionally, the goals of the participants differed from those in earlier studies. Here, the participants sought to comprehend papers describing experimental methods and results, often by comparing unfamiliar methods to known, more familiar ones. To accomplish these goals, perhaps a more balanced and diverse set of analogies is most effective.

Another possibility for explaining the balance between within- and outside-domain analogies that we observed is concerns the economics discipline itself. In Dunbar's (1995, 1997) biology lab observations, the overwhelming number of within-domain analogies may have stemmed from the fact that a majority of situations in molecular biology are likely to have clear relational correspondences to closely related areas within biological research, rather than remote domains comprised of non-molecular elements. Meanwhile, the Blanchette and Dunbar (2001) study of political commentary suggested that politicians and journalists appeared to draw intentionally from remote domains that would be familiar to readers in ways intended to highlight certain aspects of relational comparisons. In the present study, discussions across the relatively broad domain of economic inquiry highlighted the technical overlap between its various subdomains. Unlike molecular biology, economics has a high potential for relational alignment to more remote domains within public policy, banking, and corporate practice, domains in which human behavior, monetary valuations, and world affairs converge. Thus, the complexity of economics, which plays out both in academic analytic settings and in real-world financial markets, appears to provide a rich field optimal for both within-domain and out-of-domain analogical comparisons. Given the complicated nature of economic systems, economic analogies are rarely complete, so both superficial and structural correspondences appear to be drawn upon in order to explain and describe various aspects of complex systems. The validity or appropriateness of analogies in economics may also be subject to greater interpretation than other domains given our limitations in fully explaining human behavior and market dynamics.

In terms of a possible relationship between distance and depth, it has been suggested that within-domain analogies present more superficial similarity than distant analogies; in other words, the greater the distance, the less superficial the comparison (Christensen and Schunn, 2007). However, we found no evidence of that constraint in our observation of economists.

### Analogical purpose

It is fairly well established that goals influence the production of analogies (Dunbar, 1995, 1997; Spellman and Holyoak, 1996; Blanchette and Dunbar, 2001). Prior studies examined the goals of experts in scientific laboratories in both *discovery* (e.g., problem solving, hypothesis generation, experimental design) and *non-discovery* (e.g., explanation, illustration, or visualization) activities. The primary difference in purpose between the economics reading group and groups observed in the cited studies was the absence of discovery goals in the reading group. Since we determined that the discussions we observed were largely non-discovery in nature, we focused on categorizing the extracted analogies into groupings based on the perceived reason for selecting the particular analogy; e.g., to differentiate a concept from other concepts, to inject emotion or colorful language into a comparison, to give a concrete example of a more abstract idea, etc. The list we derived can be found in the Results section above.

Blanchette and Dunbar (2001) provided evidence that goals influence analogical production, but the goals of the individuals in their study appeared to have no effect on analogical distance. Saner and Schunn (1999), on the other hand, found that goals did impact the domain distance. In particular, they found that individuals used within-domain analogies when working to identify problems but used outside-domain analogies when explaining issues or concepts to their lab mates. In our study, too, the purpose-distance effect was significant. Exemplification was associated with within-domain analogizing, while visualization was strongly related to out-of-domain analogy use. It may be that the functions that result in within-domain analogies (i.e., problem identification, generation of concrete examples) share some of the same underlying cognitive operations, as do those that produce outside-domain analogies (i.e., explaining issues, visualizing concepts), in ways that influence the generation and retrieval of analogies.

In interpreting our results, we should mention that the categories we developed were not mutually exclusive; e.g., an analogy intended to differentiate between concepts might do so using visual elements. In cases where an analogy plausibly served multiple purposes, the raters chose the category they felt best described its purpose.

### General discussion on the richness of observed analogies

We have already emphasized that the collected passages contained a wealth of analogies rich in depth and structure, many of which involved implied systems of complex relations that could not be fully identified and analyzed. Furthermore, some of the more complex comparisons actually involved multiple analogies at different distances and depths. The challenges we faced in terms of analyzing the passages were made more difficult due to the complexities of the structural and superficial analogies used. Unlike laboratory tasks, in which comparisons between explicit statements are made (Gentner and Landers, 1985; Gentner et al., 1993; Krawczyk et al., 2004, 2005), the comparisons made by our economics experts were not always made fully explicit. Indeed, we frequently encountered instances in which a source or a target were implied, or not even articulated in the flow of discussion. This interpretive challenge may have been exacerbated by the high degree of expertise in our sample group, as some analogies involved inside knowledge that the speaker did not feel was necessary to clearly articulate in order to meaningfully draw the comparison to the group.

These observations are offered for several reasons. First, there are additional questions that can be investigated from these data, aside from the boundaries of this paper (e.g., Do the within-category/outside-category comparisons correspond to particular types of source-target pairs? Does a taxonomy of source categories emerge from the outside-category analogies in this setting? How effective did the analogies seem to be in conveying the intended information?). Second, the rating process involved some inherent subjectivity; even though inter-rater reliability was rather high, there were disagreements on specific passages. This is a challenge inherent in real-world analogical analyses, as the dynamic and unscripted nature of the interactions can produce text that is very difficult to interpret outside of the context in which it was spoken and by an individual who does not have access to the speaker's intent. Third, the rating process was further complicated when analogies were embedded in familiar language constructs—well-known clichés, common metaphors, etc.—and were not immediately identified as analogies by the raters, as such phrases have become so conventionalized that their figurative qualities can be simply overlooked.

We were able to ascertain some of the major purposes for which analogies are used in economics discussions among experts. The leading purpose for analogical comparisons was to provide examples. Good examples from other domains or familiar sources can provide clarity to a target and make the concept more concrete. In many of the analogies, we observed that experts tended to either relate an abstracted theoretical model to a more common situation that occurs in human interactions, or they did the reverse and described a particular situation as being an example of what is known to occur within a particular theoretical game. Experts also made common use of analogies to create a visual image of a particular concept. Visualization is important for providing a common ground for the audience and for making a target domain richer and easier to comprehend. Experts also used analogies in order to add color or interest to their contributions and to mark a topic as being included within a more familiar source domain. Given that we conducted this observation process in an academic setting, our experts may have developed tendencies to use analogy due to their experiences with teaching, in which good examples and visual comparisons can be useful for conveying concepts (Richland et al., 2007; Glynn, 2008). These goals for analogical comparison fit with prior observations about analogy as being diverse in function (Holyoak and Thagard, 1995), but contrary to several prior scholarly works describing analogical purpose (Holyoak and Thagard, 1997; Hummel and Holyoak, 1997) we did not find substantial evidence of new inferences being generated. Rather, the most prevalent use of analogy in the economics experts was to describe concepts to the group or to point out similarities between relational systems in either the real world, or in theoretical constructs.

In addition to these questions, several limitations with regard to the method and analysis of this study should be noted. The transcribers were non-experts in economics, but they were chosen to avoid domain bias and reduce the tendency to add their own interpretation to the passages. Additionally, three of the four transcribers lacked prior transcription experience in recognizing analogies, which opened the door for subtle analogies to be overlooked. We addressed this limitation using a two-phased training process and a two-person extraction strategy and believe that we reduced - but may not have eliminated - the likelihood of missed analogies. While it is possible that some subtle analogies may have been overlooked, we believe the number to be small enough to not alter our findings.

Their lack of domain expertise, however, may have contributed to the subjectivity and inconsistency mentioned above. Furthermore, we did not code the analogies according to any sort of standard domain taxonomy, as Dunbar (1995) did in his microbiology laboratory study. Finally, we did not account for individual differences by examining patterns of analogy use by specific individuals in the group.

### Conclusions

In summary, this study reinforces the strong reliance humans place on analogies for developing understanding and communicating in natural settings. It contributes valuable evidence that humans are quite agile in their selection of analogies, drawing on a mix of shallow and deep comparisons and determining an effective distance strategy based on the constraints of the domain and the level of perceived group expertise. Economics faculty and graduate student experts engaged in scientific discussion were observed to apply analogies that were more balanced in terms of categorical distance and structural depth than those observed in other natural settings. The domain context, problem-solving goals, and participant expertise of this particular group setting all appear to be important factors that led to differences in the magnitudes of depth and distance observed in the earlier studies. No evidence of an association between the distance and depth characteristics was found, but distance and purpose appeared to be related. A number of other questions remain to be answered about the ways in which analogies are applied in different types of settings. Future naturalistic analogy research could help to clarify the role on analogical comparisons in real-world settings and the social sciences appear to provide particularly rich domains for additional studies.

### Conflict of interest statement

The authors declare that the research was conducted in the absence of any commercial or financial relationships that could be construed as a potential conflict of interest.

## References

[B1] Ball L. J. Ormerod T. C. Morley N. J. (2004). Spontaneous analogising in engineering design: a comparative analysis of experts and novices. Des. Stud. 25, 495–508 10.1016/j.destud.2004.05.004

[B1a] Bearman C. R. Ball L. J. Ormerod T. C. (2002). An exploration of real-world analogical problem solving in novices, in Proceedings of the 24th Annual Conference of the Cognitive Science Society (Mahweh, NJ: Erlbaum).

[B2] Bearman C. R. Ball L. J. Ormerod T. C. (2007). The structure and function of spontaneous analogising in domain-based problem solving. Thinking Reasoning 13, 273–294 10.1080/13546780600989686

[B3] Blanchette I. Dunbar K. (1997). Constraints underlying analogy use in a real world context: politics, in Proceedings of the 19th Annual Conference of the Cognitive Science Society (Mahweh, NJ: Erlbaum).

[B4] Blanchette I. Dunbar K. (2000). How analogies are generated: the roles of structural and superficial similarity. Mem. Cogn. 28, 108–124. 10.3758/BF03211580 10714143

[B5] Blanchette I. Dunbar K. (2001). Analogy use in naturalistic settings: the influence of audience, emotion, and goals. Mem. Cogn. 29, 730–735. 10.3758/BF03200475 11531227

[B32] Catrambone R. (1997). Reinvestigating the effects of surface and structural features on analogical access, in Proceedings of the Nineteenth Annual Conference of the Cognitive Science Society, eds Shafto M. G. Langley P. (Mahwah, NJ: Erlbaum), 90–95.

[B6] Christensen B. T. Schunn C. D. (2007). The relationship of analogical distance to analogical function and preinventive structure: the case of engineering design. Mem. Cogn. 35, 29–38. 10.3758/BF03195939 17533877

[B7] Crano W. D. Brewer M. B. (2002). Principles and Methods of Social Research. Mahwah, NJ: Erlbaum.

[B8] Dunbar K. (1995). How scientists really reason: scientific reasoning in real-world laboratories, in The Nature of Insight, eds Sternberg R. J. Davidson J. E. (Cambridge, MA: MIT Press), 365–395.

[B9] Dunbar K. (1997). How scientists think: online creativity and conceptual change in science, in Conceptual Structures and Processes: Emergence, Discovery and Change, eds Ward T. B. Smith S. M. Vaid S. (Washington, DC: American Psychological Association), 461–493.

[B10] Dunbar K. (2001). The analogical paradox: why analogy is so easy in naturalistic settings, yet so difficult in the psychology laboratory, in Analogy: Perspectives from Cognitive Science, eds Gentner D. Holyoak K. J. Kokinov B. (Cambridge, MA: MIT Press), 313–334.

[B10a] Forbus K. Gentner D. Everett J. Wu M. (1997). Towards a computational model of evaluating and using analogical inferences, in Proceedings of the 19th Annual Conference of the Cognitive Science Society (Hillsdale, NJ: Erlbaum).

[B11] Gentner D. (1983). Structure mapping: a theoretical framework for analogy. Cogn. Psychol. 7, 155–170. 1644282

[B12] Gentner D. Landers R. (1985). Analogical reminding: a good match is hard to find, in Proceedings of the International Conference on Systems, Man, and Cybernetics (Tucson, AZ), 607–613.

[B13] Gentner D. Markman A. B. (1997). Structure mapping in analogy and similarity. Cogn. Psychol. 25, 524–575.8243045

[B14] Gentner D. Ratterman M. Forbus K. (1993). The roles of similarity in transfer: separating retrievability from inferential soundness. Am. Psychol. 52, 45–56. 8243045 10.1006/cogp.1993.1013

[B14a] Gick M. L. Holyoak K. J. (1980). Analogical problem solving. Cogn. Psychol. 12, 306–355.

[B15] Gick M. L. Holyoak K. J. (1983). Schema induction and analogical transfer. Cogn. Psychol. 15, 1–38.

[B16] Glynn S. M. (2008). Making science concepts meaningful to students: teaching with analogies, in Four Decades of Research in Science Education: From Curriculum Development to Quality Improvement, eds Mikelskis-Seifert S. Ringelband U. Brückmann M. (Münster: Waxmann), 113–125.

[B17] Guth W. Schmittberger R. Schwarze B. (1982). An experimental analysis of ultimatum bargaining. J. Econ. Behav. Organ. 3:367.

[B18] Hofstadter D. (2001). Analogy as the core of cognition, in Analogy: Perspectives from Cognitive Science, eds Gentner D. Holyoak K. J. Kokinov B. (Cambridge, MA: MIT Press), 499–538.

[B19] Holyoak K. J. Gentner D. Kokinov B. N. (2001). Introduction: the place of analogy in cognition, in Analogy: Perspectives from Cognitive Science, eds Gentner D. Holyoak K. J. Kokinov B. (Cambridge, MA: MIT Press), 1–19.

[B20] Holyoak K. J. Koh K. (1987). Surface and structural similarity in analogical transfer. Mem. Cogn. 15, 323–340. 3670053 10.3758/bf03197035

[B22] Holyoak K. J. Thagard P. (1995). Mental Leaps: Analogy in Creative Thought. Cambridge, MA: MIT Press.

[B23] Holyoak K. J. Thagard P. (1997). The analogical mind. Am. Psychol. 52, 35–44. 9017931

[B24] Hummel J. E. Holyoak K. J. (1997). Distributed representations of structure: a theory of analogical access and mapping. Psychol. Rev. 104, 427–466.

[B25] Krawczyk D. C. Holyoak K. J. Hummel J. E. (2004). Structural constraints and object similarity in analogical mapping and inference. Thinking Reasoning, 10, 85–104 10.1080/13546780342000043

[B26] Krawczyk D. C. Holyoak K. J. Hummel J. E. (2005). The one-to-one constraint in analogical mapping and inference. Cogn. Sci. 29, 29–38. 10.1207/s15516709cog0000_27 21702794

[B27] Polya G. (1957). How to Solve it. Princeton, NJ: Princeton University Press.

[B28] Richland L. E. Zur O. Holyoak K. (2007). Cognitive supports for analogies in the mathematics classroom. Science 316, 1128–1129. 10.1126/science.1142103 17525320

[B29] Saner L. Schunn C. (1999). Analogies out of the blue: when history seems to retell itself, in Proceedings of the 21st Annual Conference of the Cognitive Science Society (Mahwah, NJ: Erlbaum).

[B30] Spellman B. A. Holyoak K. J. (1996). Pragmatics in analogical mapping. Cogn. Psychol. 31, 307–346. 8975685 10.1006/cogp.1996.0019

[B31] Wharton C. M. Holyoak K. J. Downing P. E. Lange T. E. Wickens T. D. Melz E. R. (1994). Below the surface: analogical similarity and retrieval competition in reminding. Cogn. Psychol. 26, 64–101.

