# Supplementary material for: Expert analogy use in a naturalistic setting
Source: Front Psychol. 2014 Nov 26;5:1333. doi: 10.3389/fpsyg.2014.01333 (PMC4244599; doi:10.3389/fpsyg.2014.01333)
Supplement: Supplementary file 1 [file DataSheet1.PDF]

Supplementary Material

Expert analogy use in a naturalistic setting

Donald R. Kretz[1], Daniel C. Krawczyk[1,2] *

[1]School of Behavioral and Brain Sciences, The University of Texas at Dallas, Richardson, TX, USA.
[2]Department of Psychiatry, University of Texas Southwestern Medical Center, Dallas, TX, USA.

*** Correspondence:** Dr. Daniel C. Krawczyk, The University of Texas at Dallas, 800 W. Campbell Road, GR41, Richardson, TX, 75083, USA.
daniel.krawczyk@utdallas.edu

1. Supplementary Data

1.1. Appendix A: Examples of Transcribed Analogy and Non-Analogy Passages

And that seemed to be what he was saying: “oh, the high variance one is less risky because it's a better investment,” and that's just not what we mean by risk.

Well, one of the things is that the probability for the uncertainty role is chosen by the computer and I just don't know why things chosen by the computer can create the ambiguity. It's the same result as the ambiguity aversion where you let something weird select for you.

So what you end up with is a utility function that behaves differently depending on whether you're above or below the other guy.

So the types are parameter sets, parameter combinations.

Remember the first mixture model paper we read? Their estimation technique was to estimate the proportions. So here what they've done is estimate the parameters - each individual - and then classified the individuals based on their parameter estimates and that's where they get their proportions.

In Stahl-Wilson, they almost divided the parameter space into types so each type is restricted to what their parameters can be. Here they just kinda let the parameters - you know, the type - be whatever comes from the sky and see if it corresponds to what they thought.

(Well what do you mean they don't do what they're supposed to do?) It means that there is a whole literature that you could probably stack pretty high. So you get stacks of literature and then we put them together and ran it in the lab and it doesn't fit the literature... we couldn't explain why.

It's kind of like you do something for 15 years and someone comes along and does the same thing and they're famous.

But in this particular case, they had a subject who said they were making a decision in case they were the recipient, which is like writing down on the paper: “I am confused.”

We read the two inequality aversion papers, one which had a kink in it and the other was smooth. Well, this one takes the kinked inequality aversion one and adds to it something else.

Dale has the idea of a level playing field. If people consider the field to be more level, they will behave more in their own self-interest.

Academia works like this: if you don't fit the literature, you better explain why or get out of the game.

Playing a lot of games at once is making them do something different so that we don't come out with the level playing field.

When we did the games, we just make trees for them; and when you present all the games in tree format, most people sort of condition their heads to just choose the branch of the tree that just has the highest number in it for them.

When we did the games, we just make trees for them; and when you present all the games in tree format, most people sort of condition their heads to just choose the branch of the tree that just has the highest number in it for them.

There are a lot of experimentalists that, if you walk into their office, you see all these toys like bingo cages, Dungeons and Dragons.

The one thing about econometrics is it's very magical sometimes.

It's kind of like, what do you call it, a threshold model.

You know those plastic Easter Eggs with the different candy in them at Easter? We put good marbles and bad marbles in Easter eggs and had people pull an egg out and sell it back to us for a certain price, essentially value the lottery of Easter eggs.

So expected values is the weighted values.

You know, men are the hunters, women stay home and gather and take care of babies, it makes them risk averse… even women in BA students are more risk averse than men in BA students.

What they say is: people make mistakes and so the mu is like an error term, it's like a mistake range.

Is it just better because there is more space to soak up variance? Is it like adding parameters to a regression model?

Experiment can be used for looking at wind tunnel testing on a policy or something.

It seems to me more like it's a pattern of mistake making rather than an honest to goodness caring about probabilities.

No, if it's all freckled, the probability of picking it is a handful. If the behavior is just random, the probability is just a half.

It’s a common behavior referred as the S Shape function, similar to gambling.

This noise is like mistakes or misevaluations. So in the Holten-Moyer paper, what they say is that people make mistakes because the mu is like an error term, it’s a mistake rate. People make mistakes in their evaluations of the gambles and so it makes their choice random when they make these mistakes.

Is it better just because there’s more space to soak up more variance or is it just because there’s more

parameters; is it like adding parameters to a regression model?"

So the easiest way to test a model against another model is to restrict some of the parameters to 0. That's called a nested comparison.

Most players are not that certain about God being on their side.

It's a good Bob/naughty Bob analogy.

The trend is your friend.

2. Supplementary Figures and Tables
